# Supplementary figures and images for: Major adverse cardiovascular events and hyperuricemia during tuberculosis treatment
Source: PLoS One. 2023 Nov 16;18(11):e0294490. doi: 10.1371/journal.pone.0294490 (PMC10653473; doi:10.1371/journal.pone.0294490)

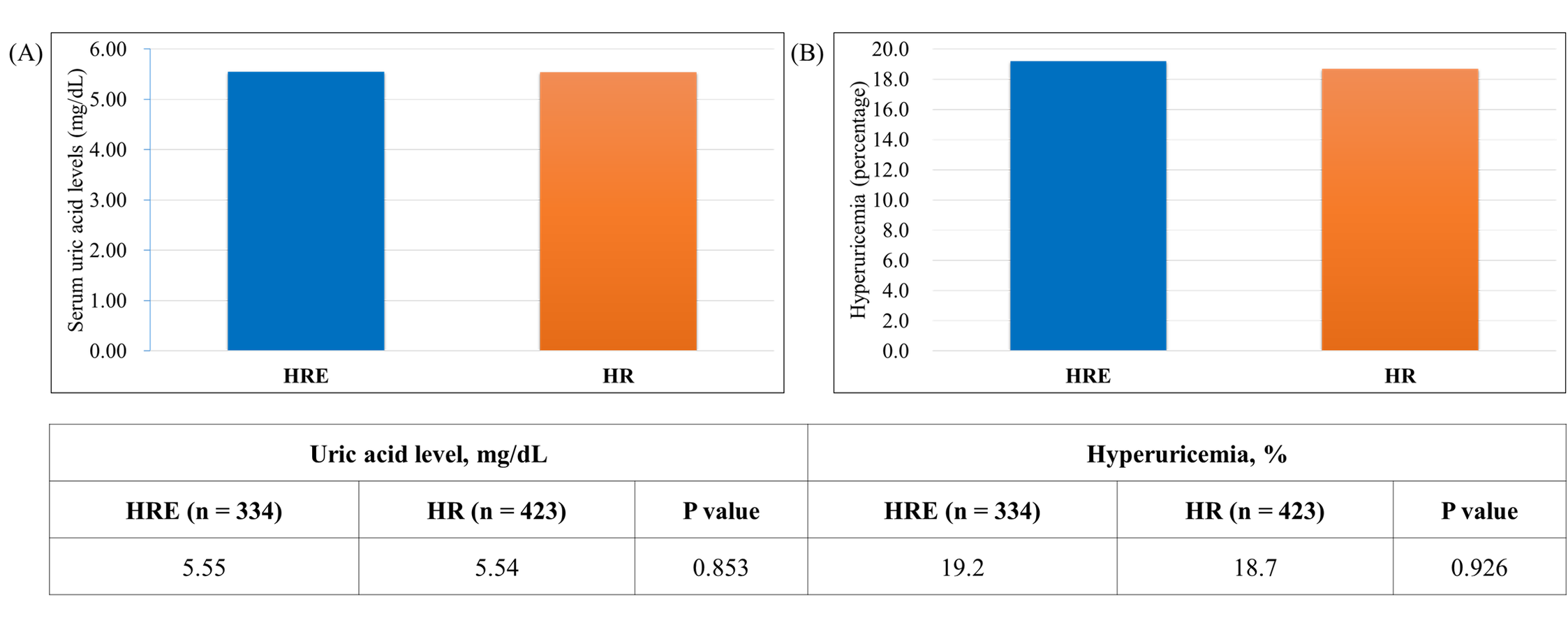

Supplement: S1 Fig — (TIF) [file pone.0294490.s002.tif]

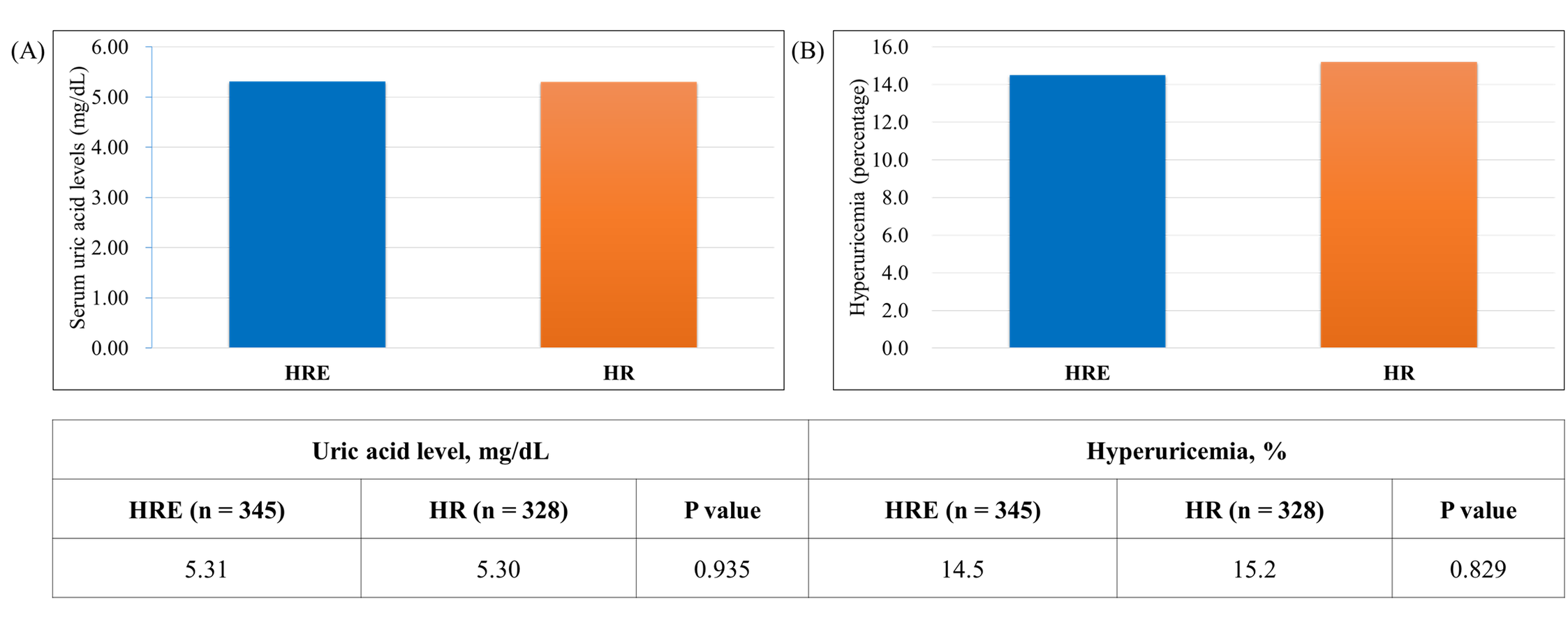

Supplement: S2 Fig — (TIF) [file pone.0294490.s003.tif]
